# Reduced Threshold for Induction of LTP by Activation of Dopamine D1/D5 Receptors at Hippocampal CA1–Subiculum Synapses

**DOI:** 10.1371/journal.pone.0062520

**Published:** 2013-04-23

**Authors:** Elisabeth Roggenhofer, Pawel Fidzinski, Oded Shor, Joachim Behr

**Affiliations:** 1 Department of Psychiatry and Psychotherapy, Charite, Universitätsmedizin Berlin, Berlin, Germany; 2 Johannes Mueller Institute of Physiology, Charite,Universitätsmedizin Berlin, Berlin, Germany; 3 Max Planck Institute for Human Cognitive and Brain Sciences, Leipzig, Germany; 4 Department of Psychiatry, Psychotherapy and Psychosomatic, Ruppiner Kliniken, Neuruppin, Germany; Federal University of Rio de Janeiro, Brazil

## Abstract

The phasic release of dopamine in the hippocampal formation has been shown to facilitate the encoding of novel information. There is evidence that the subiculum operates as a detector and distributor of sensory information, which incorporates the novelty and relevance of signals received from CA1. The subiculum acts as the final hippocampal relay station for outgoing information. Subicular pyramidal cells have been classified as regular- and burst-spiking neurons. The goal of the present study was to study the effect of dopamine D1/D5 receptor activation on synaptic transmission and plasticity in the subicular regular-spiking neurons of 4–6 week old Wistar rats. We demonstrate that prior activation of D1/D5 receptors reduces the threshold for the induction of long-term potentiation (LTP) in subicular regular-spiking neurons. Our results indicate that D1/D5 receptor activation facilitates a postsynaptic form of LTP in subicular regular-spiking cells that is NMDA receptor-dependent, relies on postsynaptic Ca^2+^ signaling, and requires the activation of protein kinase A. The enhanced propensity of subicular regular-spiking cells to express postsynaptic LTP after activation of D1/D5 receptors provides an intriguing mechanism for the encoding of hippocampal output information.

## Introduction

Cornu ammonis (CA) 1 pyramidal cells receive direct sensory information from the cortex, as well as sequential phase-precession information from the dentate gyrus (DG)–CA3 network. CA1 pyramidal cells may, thus, detect mismatches between the information coding of predictions from the DG–CA3 network that is relayed through the Schaffer collaterals and actual sensory input from the cortex. This detection of discrepancies implies that it is possible to estimate predictions for new information [1], [2]. Although CA1, in particular, is often implicated in detection and comparison of the sensory information accounting for the signal novelty [1]–[3], this function has also been attributed to the subiculum [4], [5]. The subiculum appears to receive and compare at least three different inputs of sensory associative signals that originate from the peri- and postrhinal cortices, entorhinal cortex, and CA1 [4], [6]. The subiculum seems to operate as a detector and distributor of sensory information, which incorporates the novelty and relevance of signals received from CA1 [4], [7]. There is strong evidence that the subiculum acts as the final major hippocampal relay station within the hippocampus – ventral tegmental area (VTA) loop for the unidirectional outgoing information, that seems to control the input of novel sensory information into long-term memory [8]. Time-locked release of dopamine in the hippocampal formation likely allows for the encoding of novel information [1], [9]–[11].

Activity-dependent synaptic plasticity, in particular long-term potentiation (LTP), is regarded as one of the cellular mechanisms of learning and memory [12]–[14]. The induction and expression of the early form of LTP in CA1 is modulated by activation of D1/D5 receptors and partially inhibited by D1/D5 receptor antagonism [10], [15], [16]. In addition, D1/D5 receptor activation promotes the persistence of a late (beyond 2–4 hours after activation) protein synthesis-dependent phase of LTP in CA1 [17]–[19]. A major difference between CA1 and subicular pyramidal neurons resides in their discharge behavior. While the majority of CA1 pyramidal neurons show regular-spiking behavior in response to depolarizing current injection [20], subicular pyramidal cells are characterized by two distinct discharge patterns: burst-spiking cells that display high-frequency bursts of action potentials and regular-spiking cells that discharge in a regular pattern of continuously decreasing frequency, comparable to CA1 pyramidal neurons [21]–[25]. In a previous study, we showed that activation of D1/D5 receptors lowers the threshold of LTP induction in subicular burst-spiking neurons, but not in CA1 neurons, when using a stimulation protocol that was set at a level that was subthreshold for LTP induction [26]. Our results indicated that, compared to CA1, subicular burst-spiking neurons show a very high sensitivity to express LTP after activation of D1/D5 receptors. In subicular burst-spiking cells, activation of D1/D5 receptors boosts a presynaptic form of LTP that is NMDA receptor-dependent and that requires the activation of protein kinase A (PKA). In the present study, we demonstrate that stimulation of D1/D5 receptors also reduces the threshold of LTP induction in subicular regular-spiking neurons. However, in contrast to subicular burst-spiking cells, in regular-spiking cells activation of D1/D5 receptors primes a postsynaptic NMDA receptor-dependent form of LTP.

## Materials and Methods

All procedures were performed in strict accordance with national and international guidelines [27] and were approved by the Regional Berlin Animal Ethics Committee. All efforts were made to minimize suffering. Wistar rats (4–6 weeks old) of both sexes were decapitated under deep ether anesthesia and the brains were quickly removed. 400 µm thick horizontal slices containing the hippocampus and the entorhinal cortex were prepared with a vibratome (Campden Instruments, Loughborough, UK). The preparation was performed in different preparation solutions depending on the subsequent type of recording. For intracellular recordings with sharp microelectrode, a physiological, ice-cold, oxygenated (95% O_2_, 5% CO_2_) artificial cerebrospinal fluid (ACSF) was used and the slices were transferred to an interface recording chamber for storage. The chamber was continuously perfused (1.5–2 ml/min) with oxygenated and prewarmed (34°C±0.5) ACSF. The composition of ACSF was as follows (in mM): NaCl 129, Na_2_PO_4_ 1.25, NaHCO_3_ 26, KCl 3, CaCl2 1.6, MgSO_4_ 1.8, and glucose 10 at a pH of 7.4. For patch-clamp recordings, slices were prepared in ice-cold, saccharose-based ACSF, composed as follows (in mM): NaCl 87, Na_2_PO_4_ 1.25, NaHCO_3_ 26, KCl 2.5, CaCl_2_ 0.5, MgCl_2_ 7, saccharose 75, and glucose 25 at a pH of 7.4. After preparation, slices were initially kept under submerged conditions at 34°C for ∼30 min, and were then transferred to a physiological ACSF solution at room temperature for submerged storage. Patch-clamp recordings were performed under submerged-conditions with oxygenated and pre-warmed (34°C±0.5) ACSF.

**Figure 1:**
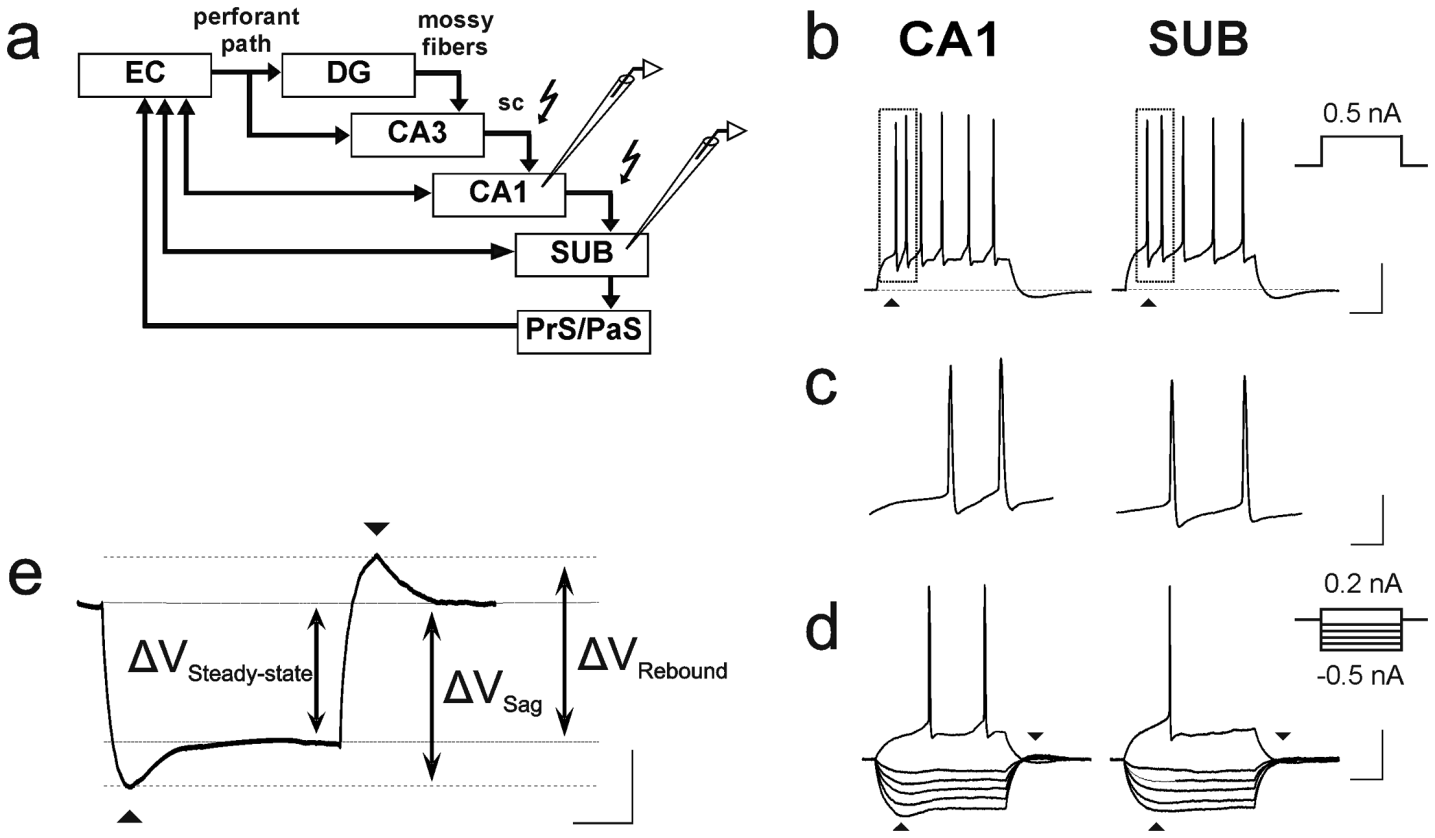
Stimulation site and electrophysiological properties of subicular regular-firing cells in hippocampal formation. **a**) Schematic illustration of hippocampal formation with stimulating and recording electrodes in SUB. Arrows mark excitatory connections, a flash marks the stimulation site in the alveus, between CA1 and SUB. Hippocampal areas: DG  =  dentate gyrus, CA1, CA3, SUB  =  subiculum, EC  =  entorhinal cortex, sc  =  schaffer collaterals. **b**) Voltage responses upon depolarizing and hyperpolarizing current pulses. Cells discharged with single spikes, each followed by a fast afterhyperpolarization (▴). Scale bars: 25 mV, 50 ms. **c**) Expanded time scale. Initial spike frequencies and afterhyperpolarization. Scale bars: 25 mV, 10 ms. **d**) Sag (▴) and rebound potentials (▾) upon hyperpolarizing current pulses. Scale bars: 25 mV, 50 ms. **e**) Exemplary voltage response upon hyperpolarizing current pulse, illustrating calculation of sag and rebound potentials (see Methods).

Single cell recordings in the pyramidal cell layer of the subiculum (Fig. 1a) were performed at near physiological temperatures (34°C) with sharp microelectrodes (50–90 M) filled with 2.5 M potassium acetate or with patch-clamp electrodes (4–6 M). Patch-clamp electrodes were filled with (in mM): K-gluconate 135, KCl 20, HEPES 10, phosphocreatine 7, Mg-ATP 2, Na-GTP 0.3, and EGTA 0.2 and adjusted with KOH to a pH of 7.2. Access resistance did not exceed 20 MΩ and varied less than 20% during the course of the experiment. No series resistance compensation was used. To study synaptic plasticity at glutamatergic synapses, all experiments were performed in the presence of bicuculline (5 µM) or SR-95531 (gabazine, 1 µM) to block GABA_A_ receptor-mediated responses. Previous studies have demonstrated the existence or recurrent connections within the subicular network [28], [29] that frequently cause polysynaptic responses upon repetitive stimulation during GABA_A_ receptor blockade. In order to prevent polysynaptic responses, concentrations of MgSO_4_ and CaCl_2_ were elevated to 4 mM each [30]–[32]. This condition has no obvious effect on the induction and expression mechanism of LTP in subicular pyramidal cells [33] or in CA1 pyramidal cells [26].

For characterization of intrinsic discharge and membrane properties, hyper- and depolarizing current steps (−0.5 to 0.5 nA, 200 ms) were used. During a hyperpolarizing current pulse of −300 pA, sag potentials were estimated as differences between the resting membrane potential and the negative peak membrane voltage during hyperpolarization (1^st^ arrow, Fig. 1e). Rebound potentials were evaluated as differences of membrane potentials before and after the end of the hyperpolarizing current pulse (2^nd^ arrow, Fig. 1e), using the local minimum of the membrane potential. Sag ratio and rebound ratio were calculated by dividing the steady-state voltage during the hyperpolarizing current pulse by sag and rebound potential, respectively (Fig. 1e). Excitatory postsynaptic potentials/currents (EPSPs/EPSCs) were evoked by stimulation of the alveus between CA1 and the subiculum. The stimulus intensity was set between 1.5–4 V, after adjusting the EPSP/EPSC amplitudes to 30–40% of the maximum response to prevent action potential discharges during the course of the experiment. Baseline responses were recorded at 0.033 Hz for at least 10 min. Based on previous studies [26] in which the total number of pulses and their frequency were systematically adjusted, the high-frequency stimulation (HFS) protocol was set at a level subthreshold for LTP induction (sharp microelectrode recordings: 25 pulses at 50 Hz; whole-cell patch-clamp recordings: 100 pulses at 50 Hz) in all experiments.

Signals were low-pass filtered at 3 kHz and sampled at 10 kHz by an ITC-16 interface (Instrutech Corp., Great Neck, NY, USA). Sharp microelectrode recordings were processed using TIDA software (HEKA GmbH, Lambrecht, Germany) and patch-clamp recordings were sampled using a Digidata 1440 interface and processed using PClamp10 software (Molecular Devices, Sunnyvale, CA, USA). Data points were binned from two consecutive EPSP/EPSC responses. The paired-pulse index (PPI) was investigated by analyzing the ratio of the second to the first synaptic response amplitude (EPSP2/EPSP1). The coefficient of variation (CV) was calculated as CV  =  standard deviation of EPSP/mean of EPSP and respectively CV^−2^  =  (mean of EPSP/standard deviation of EPSP)^2^, for a period of 10 min, beginning at time points 30 min before and 20 min after HFS. Statistical analysis was performed by applying Student's *t*-tests (paired and unpaired) for comparison of two time intervals or groups and by one-way analysis of variance (ANOVA) for multiple comparisons, where appropriate (SPSS, SPSS Inc., USA). Unless otherwise mentioned, the time intervals used were as follows: 10 min of baseline recordings (arithmetic mean used for normalization), at 2 min after HFS (posttetanic potentiation, PTP), and at the interval between 20 and 30 min after HFS (LTP/postHFS). LTP and postHFS values are given and represented in bar plots as arithmetic means of the normalized EPSP/EPSC/mAHP (medium afterhyperpolarization) amplitudes ± SEM (standard error of mean). Statistical significance was set to *p<0.05, **p<0.01 and ***p<0.001.

The following drugs were used: SKF 38393 ((+/−)-1-Phenyl-2,3,4,5-tetrahydro-(1H)-3-benzazepine-7,8-diol Hydrobromid), 100 µM; SCH 23390 ((R)-(+)-7-Chloro-8-hydroxy-3-methyl-1-phenyl-2,3,4,5-tetrahydro-1H-3-benzazepine hydrochloride), 10 µM; D-APV (D-2-amino-5-phosphonovaleric acid), 100 µM; H-89 (N-(2-(p-Bromocinnamylamino)ethyl)-5- isoquinolinesulfonamide dihydrochloride), 10 µM; BAPTA (1,2-bis(2-aminophenoxy)ethane-N,N,N′,N′-tetraacetic acid) 30 mM; bicuculline, 5 µM; gabazine (SR 95531 hydrobromide), 1 µM. Drugs were purchased from Sigma-Aldrich, Germany and Tocris, UK. All drugs (except SKF 38393) were applied at least 10 min prior to recording, the PKA antagonist was applied 1 hour prior to recording and throughout the entire course of the experiment (at least 30 minutes after HFS). Intracellular loading with the Ca^2+^ chelator BAPTA did not alter the spiking behavior of subicular pyramidal cells. Although some authors have previously reported BAPTA-mediated run-down of postsynaptic responses [34], in the present study stable baseline responses could be obtained in most of the recordings with BAPTA. Cells that showed a run-down of responses (∼15%) were not included. In comparison to the preceding study [26], the same slicing procedure, durations, concentrations of bath applications (including BAPTA infusion), and HFS protocols (in sharp microelectrode or whole-cell patch-clamp recordings, as described above) were used.

## Results

### Effect of D1/D5 receptor activation on synaptic and membrane properties

Recordings were conducted in regular-spiking pyramidal cells in the middle portion of the subiculum with respect to the proximo-distal axis. Subicular neurons were characterized according to their membrane properties, upon depolarizing and hyperpolarizing current injection [21], [35], [36]. The subicular regular-spiking cells displayed trains of single spikes comparable to CA1 pyramidal cells [37], each followed by a fast and slow AHP (Fig. 1b–e). The membrane properties of the subicular regular-discharging cells are summarized in Table 1. The subicular regular-spiking cells showed similar cell characteristics compared to CA1 cells [26] (Fig. 1d, Table 1).

**Table 1:** Passive and active properties of SUB regular-firing neurons.

rmp (mV)	−68.8±0.8
R _input_ (MΩ)	68.0±4.3
AP amplitude (mV)	75.5±1.4
AP half-amplitude width (ms)	1.08±0.05
sag ratio	0.93±0.02
rebound ratio	0.93±0.01
*n*	34

Note. rmp  =  resting membrane potential, R_input_  =  input resistance, AP  =  action potential.

**Figure 2:**
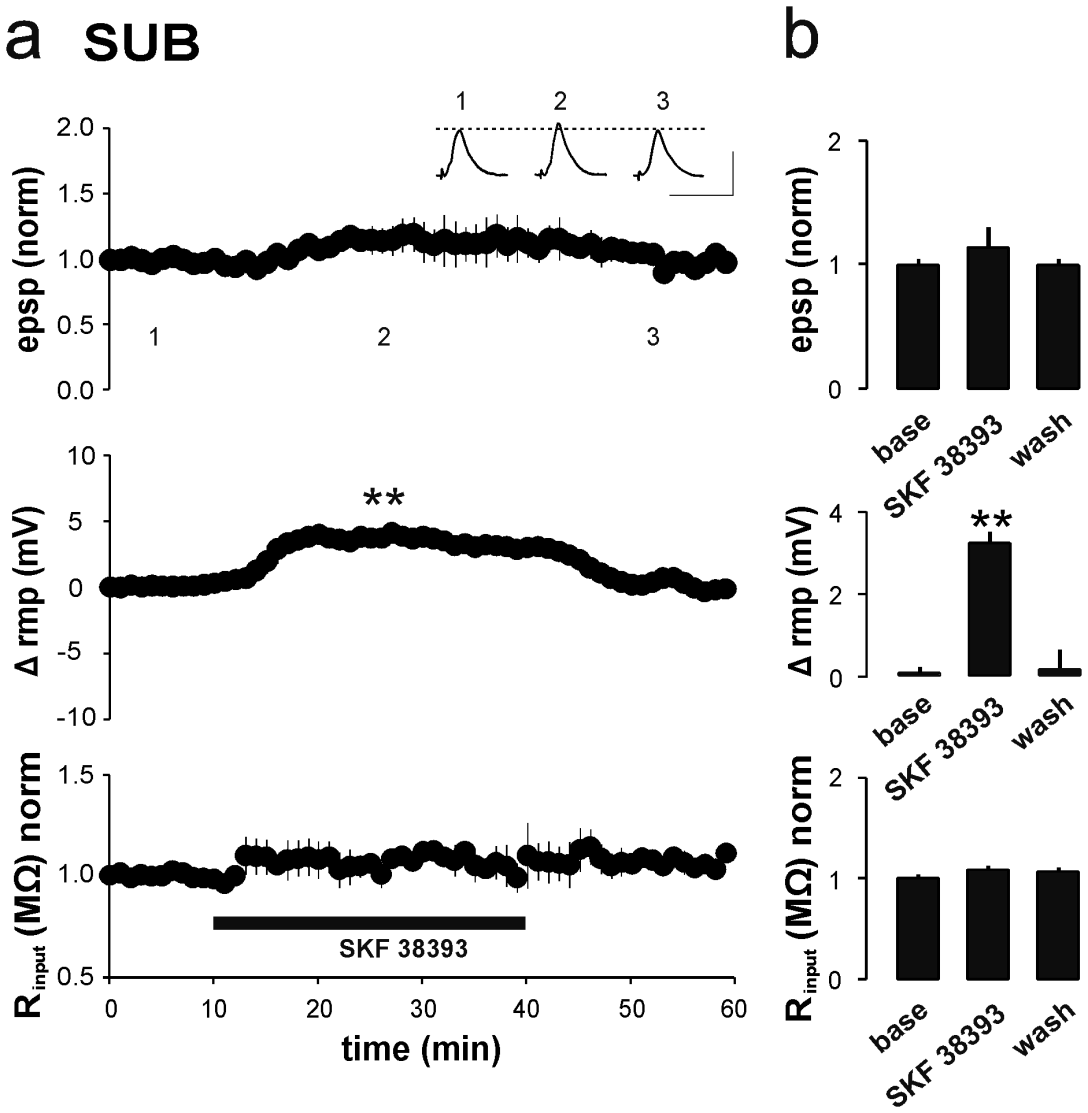
Effect of D1/D5 receptor activation on synaptic and membrane properties of SUB pyramidal cells (sharp microelectrode). **a**) Averaged time course of normalized EPSP amplitude, resting membrane potential (rmp) and input resistance (R_input_), under sharp microelectrode condition for SUB regular-firing cells at bath application of SKF 38393. During application of SKF 38393, subicular pyramidal cells showed a transient depolarization (3.20±0.23 mV, *p*<0.01, *n* = 7) that decayed to baseline level within 5 minutes after wash-out. No significant change in baseline transmission or R_input_ was observed during or 20 min after application of SKF 38393. rmps are expressed as differences to baseline rmp. Scale bars: 5 mV, 50 ms. **b**) Summary of D1/D5 receptor-mediated modulation of baseline transmission and passive membrane properties. Columns represent mean + SEM before (min 0–10), during (min 30–40) and after (min 50–60) application of SKF 38393.

As activation of D1/D5 receptors has been reported to induce an activity-independent LTP [17], [19], [38] (see also [19], [39]), we first studied whether prolonged bath application of SKF 38393 (100 µM) for 30 min affects basal synaptic transmission at CA1–subiculum synapses. Recordings were performed with sharp microelectrodes in order to leave the intracellular environment unaffected. SKF 38393 did not induce LTP in the subicular regular-spiking cells, neither during nor 20 min after application (SKF 38393: 1.14±0.16, *p*>0.05; wash-out: 1.00±0.04, *p*>0.05, *n* = 7; Fig. 2a and b). The D1/D5 receptor activation caused a small, but significant depolarization of the resting membrane potential (interval min 20–30 during application of SKF 38393, directly before wash-out: 3.20±0.23 mV, *p*<0.01) that returned to baseline levels within, on average, 5 min after wash-out (interval min 10–20 after start of wash-out: 0.13±0.43 mV, *p*>0.05). No significant change in input resistance was observed.

### Activation of D1/D5 receptors facilitates induction of LTP

**Figure 3:**
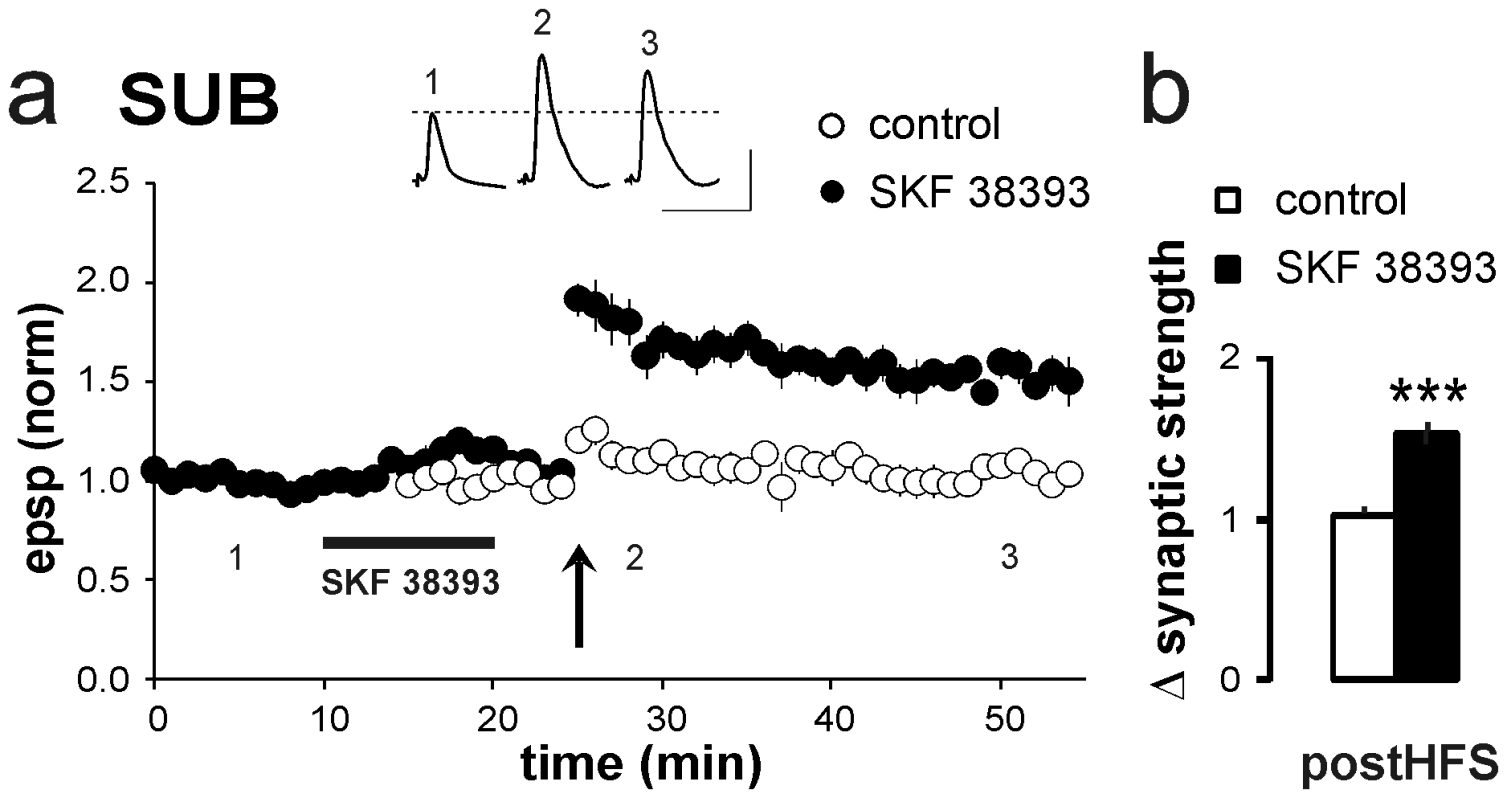
Activation of D1/D5 receptors facilitates the induction of LTP in SUB regular-firing cells. **a**) To investigate the facilitation of LTP by the D1/D5 receptor agonist SKF 38393, a brief HFS protocol was used (arrow, also see Methods), which was insufficient to induce LTP under control conditions (1.03±0.04, *n* = 10). HFS given 5 minutes after application of SKF 38393 induced a robust LTP in SUB regular-firing cells (1.53±0.08, *p*<0.001, *n* = 7). EPSPs were recorded before (1) and after HFS (2,3). Scale bars: 5 mV, 50 ms. **b**) Summary of SKF 38393-induced effects on LTP induction in SUB regular-firing neurons.

For activation of D1/D5 receptors, the specific agonist SKF 38393 (100 µM) was bath-applied for 10 min. SKF 38393 induced a slight, but not significant, increase in EPSP amplitudes and depolarization of the resting membrane potential in the subicular regular-spiking cells. Both values returned to baseline levels within 5–8 min after wash-out of the agonist. To exclude any unspecific effects due to changes in EPSP amplitude and resting membrane potential, only cells that showed a reversal of both parameters after wash-out of SKF 38393 were included in this study. To assess the wash-out kinetic of the D1/D5 receptor agonist, we analyzed the suppressive effect of the agonist on mAHPs and its reversal upon wash-out. As illustrated in figure S1, after wash-out, mAHPs recovered to baseline level. Five minutes after wash-out of SKF 38393, we applied a brief HFS protocol (25 pulses, 50 Hz), which was insufficient to induce LTP at CA1–subiculum synapses under control conditions (1.03±0.04, *p*>0.05, *n* = 10; Fig. 3a and b). After priming activation of D1/D5 receptors, however, the subthreshold stimulation protocol induced a robust and stable LTP (1.53±0.08, *p*<0.001, *n* = 7; Fig. 3a and b).

**Figure 4:**
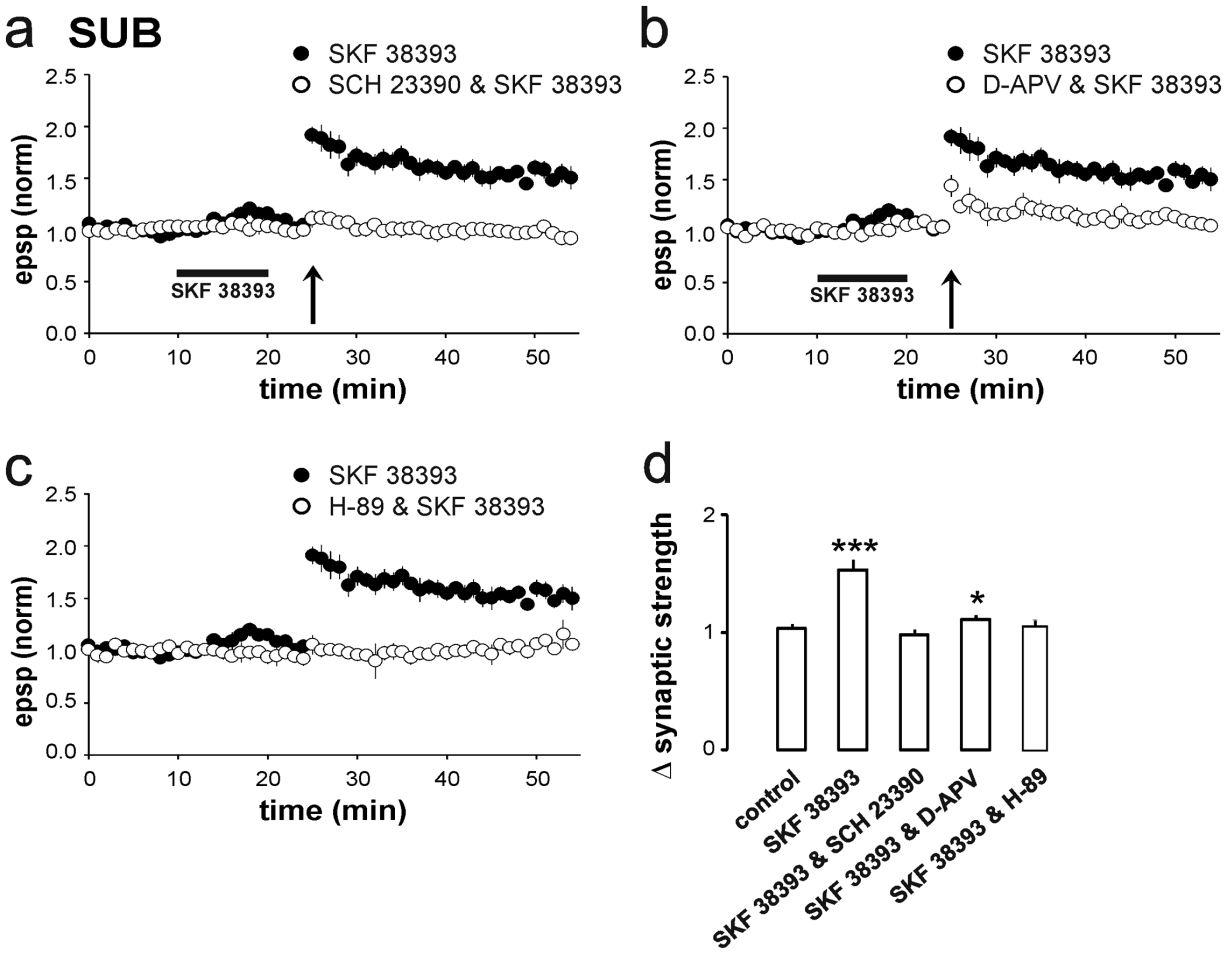
Mechanisms of D1/D5 receptor-facilitated induction of subicular LTP. **a**) HFS-induced, D1/D5 receptor-dependent LTP was blocked by the D1/D5 receptor antagonist SCH 23390 (0.97±0.05, *n* = 5). **b**) The NMDA receptor antagonist D-APV significantly reduced HFS-induced, D1/D5 receptor-dependent LTP (1.11±0.04, *p*<0.05, *n* = 5). **c**) PKA inhibitor H-89 blocked D1/D5 receptor-facilitated LTP (1.05±0.06, *n* = 5). **d**) Summary of changes in synaptic strength as illustrated in **a–c**. One-way ANOVA revealed a significant difference between the group of SKF 38393-mediated LTP and all other groups of pharmacological intervention by comparison within last 10 minutes of recordings.

We next examined whether the facilitation of LTP by SKF 38393 is mediated by D1/D5 receptor activation. In the presence of the specific D1/D5 receptor antagonist SCH 23390 [40], [41], SKF 38393 did not facilitate LTP in regular-discharging cells (0.97±0.05, *n* = 5, *p*>0.05; Fig. 4a), indicating that the facilitation by SKF 38393 is a specific D1/D5 receptor-mediated effect.

### Facilitated LTP is NMDA receptor-mediated and depends on the activation of PKA

To determine whether the facilitated induction of LTP by SKF 38393 requires the activation of NMDA receptors, we investigated the effect of NMDA receptor blockade on LTP induction. In the presence of the NMDA receptor antagonist D-APV, the SKF 38393-induced facilitation of LTP was nearly blocked (1.11±0.04, *n* = 5, *p*<0.05; Fig. 4b). Our results indicate that activation of D1/D5 receptors facilitates NMDA receptor-dependent LTP at CA1–subicular synapses. Because D1/D5 receptors are members of the G protein-coupled receptor superfamily that are positively coupled to the adenylyl cyclase–cyclic adenosine monophosphate (cAMP)–PKA cascade, we studied the SKF 38393-mediated facilitation of LTP in the presence of the PKA antagonist H-89. Hippocampal brain slices were incubated with H-89 for at least 1 hour prior to recording. In the presence of H-89, the facilitation of LTP by SKF 38393 was inhibited (1.05±0.06, *n* = 5, *p*>0.05; Fig. 4c), indicating that this form of LTP requires the activation of PKA. A summary of changes in the synaptic strength, normalized to the baseline EPSP amplitude induced by HFS, is shown in Fig. 4d. A one-way ANOVA revealed a significant difference between the groups of SKF 38393-mediated LTP and pharmacological blockade of the specific receptors and kinases (F[_4_], [_23_] = 17.81, *p*<0.001), whereas no significant difference could be observed between groups without D1/D5 receptor-mediated priming, and with inhibited induction of LTP (group comparison of control condition, SCH 23390, D-APV, H-89: F[_3_], [_17_] = 0.55, *p*>0.05).

### Facilitated induction of LTP is dependent of postsynaptic Ca^2+^

**Figure 5:**
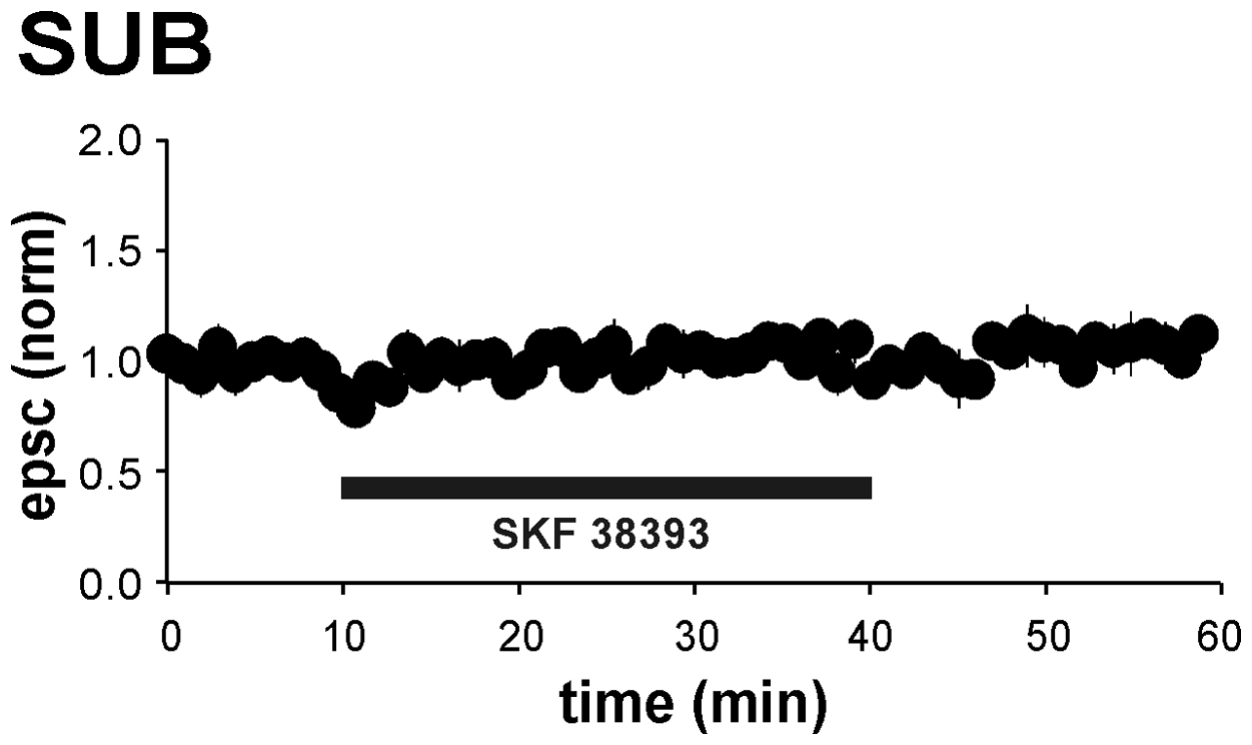
Effect of D1/D5 receptor stimulation on synaptic and membrane properties of SUB pyramidal cells (patch-clamp). Averaged time-course of normalized EPSC amplitude under patch-clamp condition for SUB regular-firing cells before, during and after bath application of SKF 38393. No significant change in baseline transmission was observed during (min 30–40: 1.04±0.07, *n* = 6) or 20 min after application of SKF 38393 (min 30–40: 1.07±0.09).

To study the role of postsynaptic Ca^2+^ signaling in the SKF 38393-induced facilitation of LTP, we performed whole-cell patch-clamp recordings in voltage-clamp mode. As is shown by the sharp microelectrode recordings (Fig. 2a and b), prolonged (30 min) bath application of SKF 38393 without HFS did not induce an increase in EPSC amplitudes, either during or up to 20 min after application (interval min 20–30 during application of SKF 38393, directly before wash-out: 1.04±0.07; interval min 10–20 after start of wash-out: 1.07±0.09, *n* = 6; Fig. 5).

**Figure 6:**
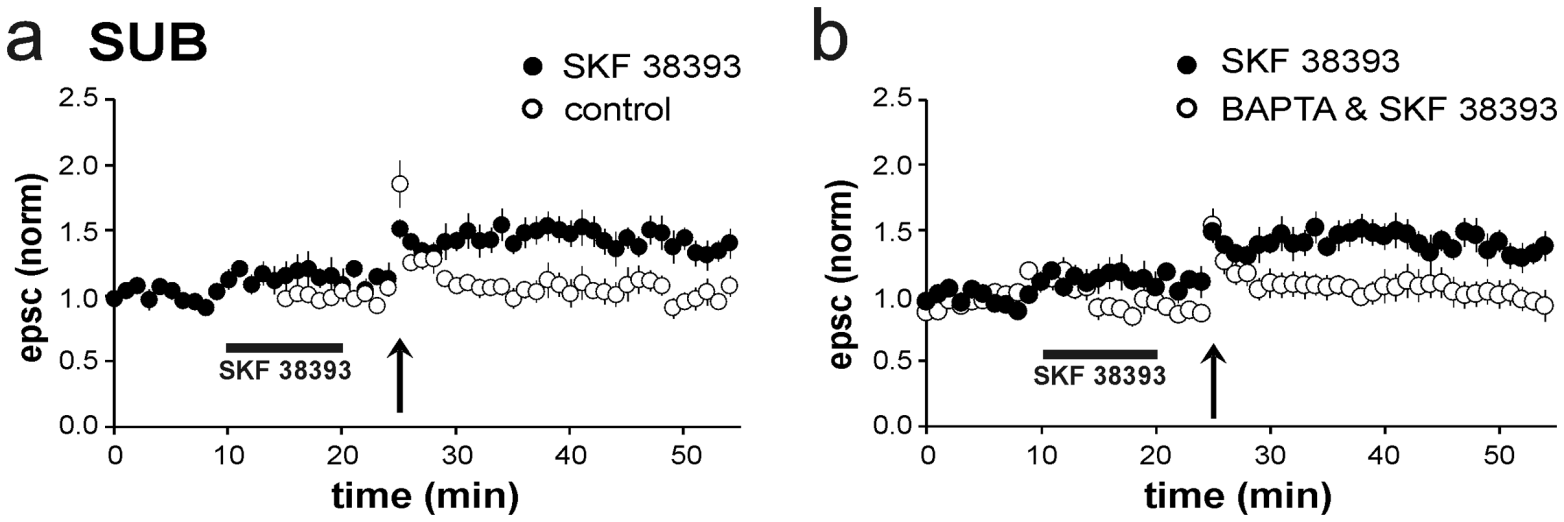
Postsynaptic Ca^2+^ signaling is required for D1/D5 receptor-facilitated LTP. **a**) HFS, which was insufficient to induce LTP on control conditions (1.03±0.09, *n* = 6), facilitated LTP after application of SKF 38393 in SUB regular-firing cells recorded in whole-cell patch-clamp technique (1.40±0.10, *p*<0.01, *n* = 8). **b**) Postsynaptic dialysis with BAPTA prevented expression of LTP after application of SKF 38393 and subsequent HFS (1.03±0.10, *n* = 5).

A subthreshold protocol (100 pulses, 50 Hz) resulted in PTP, but failed to induce LTP (PTP: 1.56±0.11, postHFS min 20–30: 1.03±0.09, *n* = 6). After application of SKF 38393, the same protocol induced LTP (1.40±0.10, p<0.01, *n* = 8; Fig. 6a). As previously shown [33], the magnitude of LTP in whole-cell patch-clamp recordings (submerged slice) is smaller than in sharp microelectrode recordings (interface condition), which might be attributed to a difference in oxygen supply [42], [43]. Individual regular-spiking cells were loaded with the fast Ca^2+^ chelator BAPTA to determine whether an increase of postsynaptic Ca^2+^ is required to facilitate LTP by D1/D5 receptor activation. Postsynaptic dialysis with BAPTA for up to 40 min before applying the subthreshold protocol prevented the induction of LTP (1.03±0.10, *n* = 5; Fig. 6b), suggesting that the SKF 38393-induced facilitation of LTP is dependent on postsynaptic Ca^2+^ signaling.

### Expression of D1?D5 receptor-mediated facilitation of LTP

**Figure 7:**
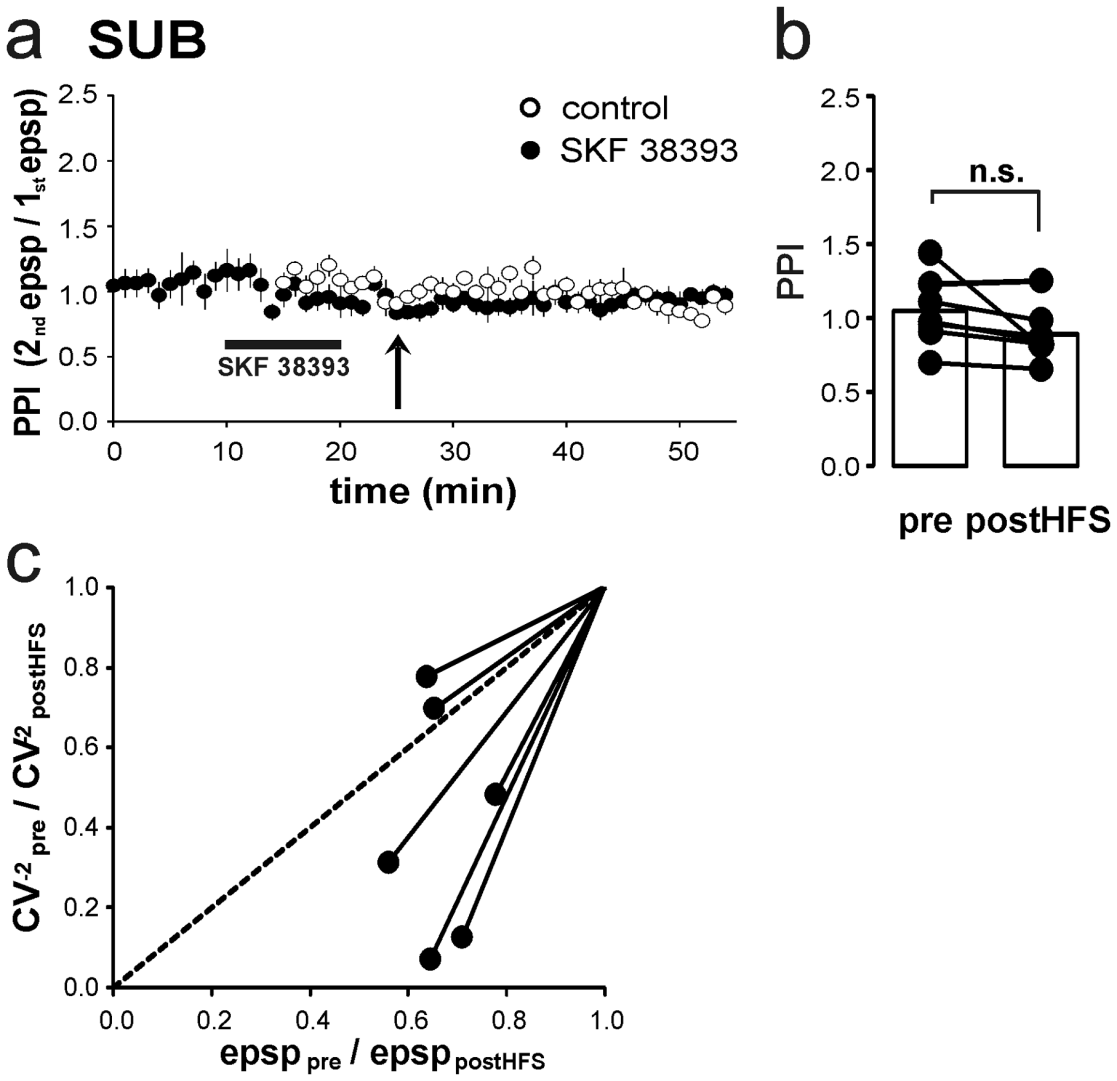
Analysis of PPI and CV indicates a postsynaptic expression of subicular D1/D5 receptor-mediated LTP. **a**) Time course of PPI without significant changes pre- (1.03±0.08) and postHFS (0.89±0.07, *n* = 7). **b**) PPI before and 20 min after induction of D1/D5 receptor-facilitated LTP. Dots represent single cells, columns mark mean (n.s.  =  not significant). **c**) Time course of CV with and without D1-/D5 receptor activation does not reveal significant changes between pre- vs. postHFS (mean CV, control – preHFS: 0.12±0.02, postHFS: 0.10±0.02, *p*>0.05, *n* = 10; mean CV, SKF 38393 – preHFS: 0.13±0.02, postHFS: 0.08±0.02, *p*>0.05, *n* = 7). **d**) CV^−2^ ratios plotted against mean EPSP ratios obtained from single cells. CV analysis indicates no increase of release probability after induction of LTP. Changes in ratio of EPSP amplitudes (0.67±0.02) are not associated with changes in ratio of CV^−2^ (0.58±0.18, *p*>0.05, *n* = 7).

One approach which has been used to address the site of LTP expression is the investigation of changes in the paired-pulse index (PPI). The PPI provides a measure with which to investigate the increase of transmitter release upon the second of two timely-spaced afferent stimuli that depends on residual Ca^2+^ levels in the presynaptic terminal [44]. The facilitated LTP was not associated with significant alterations of the PPI (SKF 38393 – PPI preHFS: 1.03±0.07 and postHFS: 0.89±0.07, *p*>0.05, *n* = 7; Fig. 7a and b). Another approach to differentiate between pre- and postsynaptic LTP expression in evoked synaptic events is the analysis of the coefficient of variation (CV) [45]. A change in the CV^−2^ indicates an increase or decrease in either transmitter release probability or in the number of functional synaptic release sites [45]–[47]. In our experiments, the induction of LTP was not associated with a significant change of the CV^(−2)^ before and after induction of LTP with and without D1/D5 receptor stimulation (mean CV, control – preHFS: 0.12±0.02, postHFS: 0.10±0.02, *p*>0.05, *n* = 10; mean CV, SKF 38393 – preHFS: 0.13±0.02, postHFS: 0.08±0.02, *p*>0.05, *n* = 7; Fig. 7c). No significant differences were observed between the averaged EPSP ratio (pre/postHFS: 0.67±0.02) and the CV^−2^ ratio (pre/postHFS: 0.58±0.18, *p*>0.05, *n* = 7; Fig. 7d). In Fig. 7d, the ratio of the CV^−2^ before and after induction of D1/D5 receptor-mediated LTP is plotted against the ratio of EPSP amplitudes. CV analysis indicated that neither the release probability nor the number of functional synaptic release sites was enhanced after induction of LTP indicative of a postsynaptic expression site. In summary, the analysis of PPI and CV do not provide evidence for a presynaptic expression of LTP in subicular regular-spiking pyramidal neurons.

## Discussion

The present study reveals that D1/D5 receptor activation facilitates a postsynaptic form of LTP in subicular regular-spiking cells that is NMDA receptor-mediated and Ca^2+^-dependent, and which requires the activation of PKA. Blockade of the postsynaptic Ca^2+^ signaling cascade by BAPTA inhibited the expression of D1/D5 receptor-facilitated LTP. In addition, LTP was not associated with a decrease of PPI or a change of the CV consistent with a postsynaptic expression of LTP.

Previous studies have demonstrated that in CA1, D1/D5 receptor activation facilitates the persistence of a late protein synthesis-dependent LTP, rather than the initial protein synthesis-independent phase [19], [26]. However, by using a suprathreshold LTP protocol, Otmakhova & Lisman [15] showed that early LTP could be enhanced by about 10–15% (30 min postHFS, dependent on the agonist) by prior activation of D1/D5 receptors. We conclude that in both CA1 and subicular pyramidal cells, synaptic plasticity can be facilitated by D1/D5 receptor activation, implying that both regions may be involved in dopamine-dependent encoding of novel information. Our previous study indicated a significantly higher sensitivity for dopamine-induced facilitation of synaptic plasticity in subicular than in CA1 pyramidal cells [26]. The distinct sensitivity of subicular pyramidal cells might be caused by either a region-specific distribution of dopamine-R subtypes, or by a different ability to effectively express LTP. Expression studies on dopamine-R subtypes have demonstrated prominent labeling of D1 receptors in the ventral subiculum, in comparison to the stratum oriens and radiatum of CA1–CA3 fields in rats [48]. For D5-Rs, strong immunostaining was found in the pyramidal cells of the hippocampus proper and of the subiculum in rats, mostly on cell bodies rather than on dendrites [49], [50]. In addition, there is some evidence that the D5 receptor is the predominant D1-like receptor in certain targets of dopaminergic innervation [49], [51]. The number of activated D1/D5 receptors may determine the difference between the LTP facilitation in CA1 and the subiculum. Therefore, in CA1, we previously used a higher concentration of SKF 38393 (500 µm) [26]. SKF 38393 entailed a slight, but not significant, depolarization during baseline transmission, but still failed to prime LTP. There is evidence that early LTP in CA1 is influenced by endogenous dopamine released by tetanic stimulation, in addition to external pharmacologic stimulation [15], [52]. However, by increasing the number of stimulation pulses (100 pulses, 50 Hz), tetanization still failed to induce LTP at CA3–CA1 synapses [26]. Taken together, these results suggest a different propensity of subicular cells to express LTP, rather than a difference in the R-distribution between CA1 and subiculum.

What might be the underlying mechanisms for the observed effects? D1/D5 receptor activation has been shown to increase voltage-gated Ca^2+^ currents [53]–[56] and decrease slowly inactivating K^+^ currents [57], both of which can affect the spiking threshold and modulate the membrane potential. Activation of D1/D5 receptors enhances the activity of adenylate cyclase, and is followed by a rise in cAMP concentration and activation of PKA [58]. PKA, in turn, is known to up-regulate NMDA receptor-mediated currents and sensitivity, via phosphorylation of the NMDA receptor subunit NR1 [59], [60]. In line with these findings, activation of D1/D5 receptors has been shown to induce a potentiation of NMDA receptor-mediated currents [38], [61]–[67]. Studies in cortical pyramidal neurons have shown that increased NMDA receptor-mediated responses are configured in an inverted U-shaped, dose-response curve [68], correlating with the finding that optimal levels of D1/D5 receptor activation are necessary for the formation of efficient working memory [69]. As the SKF 39383-induced facilitation of LTP depends on a postsynaptic Ca^2+^ signal and can be attenuated by the PKA antagonist H-89, we suggest that activation of D1/D5 receptors elevates postsynaptic cAMP concentrations and activates the cAMP-PKA cascade by a G_s_-adenylate cyclase pathway in subicular regular-spiking neurons. According to previous findings regarding area CA1 [39], [70], our results imply that activation of D1/D5 receptors does not induce a HFS-independent LTP, but can act as a permissive, priming trigger for LTP induction.

CA1–subiculum synapses express a pre- and postsynaptic form of LTP which correlates with the discharge property of the subicular pyramidal cell. The target-specificity of CA1 efferents seems to rely on two subsets of CA1 terminals, which differ in their propensity to express a presynaptic form of LTP [33], [71]–[73]. The mechanism by which CA1 terminals determine the discharge behavior of the postsynaptic subicular target cell and/or vice versa are not known yet. Depending on the subicular cell type, D1/D5 receptor activation facilitates either pre- or postsynaptic LTP. Target cell-specific mechanisms of synaptic plasticity have also been shown for hippocampal mossy fiber synapses. The properties of mossy fiber-pyramidal cell synapses are distinct from mossy fiber-interneuron synapses in terms of short-term plasticity and a presynaptic form of LTP [74], [75]. Evidence-based theories suggest that postsynaptic forms of LTP generate an unconditional gain of synaptic transmission [76]. In contrast, the presynaptic form of LTP in bursting cells might be involved in differential decoding of network activity and in altering the dynamics of synaptic transmission.

Other studies have demonstrated that activation of D1/D5 receptors is sufficient to induce a HFS-independent, protein synthesis-dependent, late form of LTP [17], [38]; a finding that could not be reproduced with the same and varying parameters concerning incubations conditions and choice of the D1/D5 receptor agonist [19], [39]. More recently, studies have indicated that D1/D5 receptor stimulation modulates, rather than induces, synaptic plasticity and that the potentiation depends on the synergistic activation of NMDA receptors by the tetanic stimulation [70]. Our results demonstrate that prolonged activation of D1/D5 receptors by SKF 38393 depolarizes the membrane potential only transiently during the application period, and is not sufficient to induce a long-lasting potentiation of synaptic transmission at CA1–subiculum synapses in regular-firing cells.

There is evidence that the subiculum is an important structure for generating novelty-dependent activation of the VTA [77]. Stimulating the subiculum with either tetanization [78], [79] or NMDA application [80], [81] causes dopamine release from VTA terminals that project to the hippocampus. In addition to CA1 [10], subicular regular-spiking cells might, hence, be regulated and modulated by transient dopaminergic and glutamatergic inputs to provide differential processing of novel and familiar sensory information from the hippocampus to different cortical and subcortical brain regions.

## Supporting Information

Figure S1 **Effect of D1/D5 receptor stimulation on mAHPs. a**) Averaged time course of normalized mAHP amplitudes. **b**) Averaged mean of normalized mAHP amplitudes shows a significant transient decline during application of SKF 38393. Columns illustrate mean + SEM before (base, min 0–5: 1.03±0.04, base vs. SKF 38393 *p*<0.05), during (SKF 38393, min 10–15: 0.93±0.04, SKF 38393 vs. wash-out *p*<0.05) and after (wash-out, min 35–40: 1.09±0.05, *n* = 7) application of SKF 38393.
(TIF)
